# Maternal and neonatal outcomes and determinants of iodine deficiency in third trimester of pregnancy in an iodine sufficient area

**DOI:** 10.1186/s12884-020-02863-6

**Published:** 2020-03-18

**Authors:** Soraya Saleh Gargari, Reyhaneh Fateh, Mina Bakhshali-bakhtiari, Masoumeh Saleh, Masoumeh Mirzamoradi, Mahmood Bakhtiyari

**Affiliations:** 1 Department of Gynecology & Obstetrics, Shohada Tajrish Educational Hospital, Shahid Beheshti University of Medical Sciences, Tehran, Iran; 2 Department of Gynecology & Obstetrics, Mahdiyeh Hospital, Shahid Beheshti University of Medical Sciences, Tehran, Iran; 3 Non-Communicable Diseases Research Center, Alborz University of Medical Sciences, Karaj, Iran; 4 Department of Community Medicine, Alborz University of Medical Sciences, Karaj, Iran

**Keywords:** Urinary iodine concentration, Maternal and neonatal complications, Pregnancy

## Abstract

**Background:**

Mild to moderate iodine deficiency in pregnant women may expose them to the increased risk of the development of goiter and thyroid disorder. There is a relationship between low maternal UIC (Urinary iodine concentration) in pregnancy and diminished placental weight and neonatal head circumference. The current study was an attempt to assess iodine nutritional status, its determinants and relationship with maternal and neonatal outcomes.

**Methods:**

In this population based cross-sectional study, which was conducted from April 2017 to September 2018, information was collected from 884 women of 20–45 years old who referred for periodic pregnancy visits. UIC was measured in random urine samples by applying a manual method which was based on the Sandell–Kolthoff technique. Information related to neonatal and maternal complications was collected from the individuals enrolled in the study through systematic follow-ups of the research team in each hospitals and the referral of trained midwives to the place of delivery and the retrieval of the case files.

**Results:**

The results showed that out of 884 participants 838 (94.8%) had a urinary iodine concentration of more than 150 micrograms/litre and 46 (5.2%) showed urinary iodine concentrations less than 150 micrograms/litre. The median (IQR) urinary iodine concentration in the third trimester was 176 (165–196) μg/l. According to the WHO criteria 46 of the participants (5.2%) had insufficient urinary iodine concentrations, 805 (91.06%) had adequate urinary iodine concentrations while 33 (3.73%) showed more than adequate levels. There were no participants with urinary iodine concentrations higher than 500 micrograms/litre. The main influencing factors on maternal iodine deficiency in this study were weight gain during pregnancy (Odds Ratio (OR) =0.88, 95% CI: 0.82–0.95), number of previous pregnancy (OR = 0.59, 95% CI: 0.39–0.89) the interval between the most recent pregnancies (OR = 0.78, 95% CI: 0.64–0.95), whether or not the pregnancy has been Planned (OR = 2.92, 95% CI: 1.29–6.58) and nutritional complement consumption (OR = 3.64, 95% CI: 1.44–9.1). The need for a neonatal intensive care unit (NICU) admission (OR = 4.64, 95% CI: 1.81–11.9) and preterm birth (OR = 3.29, 95% CI: 1.51–7.1) were significantly related with maternal iodine deficiency before delivery. Also there is no significant differences regarding the mean maternal urinary iodine concentration between the normal and different maternal complications groups (*p* = 0.47).

**Conclusion:**

Iodine deficiency in pregnant women can be improved by appreciate planning for pregnancy, proper inter-pregnancy time interval (> 12 months to < 5 years), appropriate nutrition during pregnancy. Besides, controlling maternal urinary iodine concentrations is important to prevent neonatal complications such as preterm delivery and NICU admission.

## Background

Numerous physiologic changes in the production of thyroidal hormone during pregnancy can be observed; thus, an increase in the amount of iodine intake should be considered in order to meet the higher demands which are needed for the production of thyroxine (T4), transferring iodine to the fetus, and increasing the clearance of renal iodine by the mother [1–3]. Due to the elevated production of thyroid hormone, the elevated excretion of renal iodine, and the requirements of fetal iodine, dietary iodine requirements are higher in pregnant adults than non-pregnant ones [4]. Mostly, to determine iodine status in populations, spot urinary iodine values are utilized. Since considerable diurnal and daily variations can be observed in urinary iodine excretion, urinary iodine concentrations (UICs) cannot be applied to detect specific individuals with iodine deficiency [5, 6]. Women enjoying suitable iodine intake before and during pregnancy have suitable intrathyroidal iodine stores, so they can easily adapt to the increased demand for thyroid hormone during pregnancy. Moreover, total-body iodine levels in these women will be constant during pregnancy [7]. Severe shortage of iodine in pregnant women may give rise to the increased rates of pregnancy loss, stillbirth, and increased perinatal and infant mortality or lead to fetal iodine deficiency damaging neurocognitive growth of the growing fetus [8–11].

Mild to moderate iodine deficiency in pregnant women may expose them to the increased risk of the development of goiter [12] and thyroid disorders [13]. There is a relationship between low maternal UIC in pregnancy and diminished placental weight and neonatal head circumference [14].

The current study was an attempt to assess iodine nutritional status, its determinants and relationship with maternal and neonatal outcomes.

## Method

### Study design

In this population based cross-sectional study, which was conducted from April 2015 to September 2016, information was collected from 884 women of 20–45 years old who referred for periodic pregnancy visits. The inclusion criteria were singleton pregnancy, not having diabetes, hypertensive disorders of pregnancy, and thyroid diseases, and not taking thyroid medications.

### Sampling

Firstly, a list of all hospitals and medical centers in Tehran was provided and by subsequent phone calls, those hospitals in which the gynecology department was active were entered the initial sample list. Then, the approximate location of hospitals was determined on the standard map of the city of Tehran. Since the hospital admission policies varied widely in Tehran’s 22 districts, the effect of the location and socio-economic status were considered by using a systematic random sampling approach. Finally, 6 hospitals (Mahdiyeh hospital from district 16, Emam khomeni hospital from district 6, Emam hossien hospital from district 13, Shohaday tajrish hospital from district 1, Erfan and Bahman hospitals from district 2 were chosen as sampling area) were selected in different areas of North, South, Center, West, and East of Tehran for individual sampling. Considering that the policy of admission and the number of referrals were different in these hospitals, in this study, sampling proportional to the size of admission was performed (270 sample from Mahdiyeh hospital, 340 sample from Emam khomeni hospital, 110 sample from Emam hossien hospital, 85 sample from Shohaday tajrish hospital and 84 sample were chosen from Erfan and Bahman hospitals). Sample selection within the each selected hospital was done by using simple random sampling method. In the present study, 19 individuals due to the loss or lack of urine sample, 11 individuals due to the lack of follow up of their newborn because of transmitting to other medical centers, and 24 individuals due to not cooperating with the research team and lacking data were excluded from the study. Finally, data collected from 884 individuals out of a total of 938 individuals who entered into the study were analyzed.

### Data collection procedure

In the present study, maternal basic and demographic data (including age, gestational age, weight gain during pregnancy, mother educational status, smoking, parity, gravidity, time interval between the recent pregnancy and inclusion criteria) were recorded by a trained midwife. In this study, gestational age was calculated based on the first day of the last normal menstruation. Moreover, the gestational age greater than 24 weeks was considered as third trimester of gestation. Information related to neonatal and maternal complications was collected from the individuals enrolled in the study through systematic follow-ups of the research team in each hospitals and the referral of trained midwives to the place of delivery and the retrieval of the case files. The severity of congenital hypothyroidism (CH) is classified based on initial free T4 (FT4) concentrations. we classified CH as severe (FT4 < 5 pmol/L [< 0.39 ng/dL]), moderate (FT4 5–< 10 pmol/L [0.39–< 0.78 ng/dL]), or mild (FT4 ≥ 10 [≥0.78 ng/dL]) [15].

Urinary iodine concentration (UIC) was measured in random urine samples by applying a manual method which was based on the Sandell–Kolthoff technique. The results are presented as microgram of iodine per liter of urine (μg/L). The analytical sensitivity for iodine was 1.39 μg/L and the intra-assay and interassay coefficients of variation were 4.4 and 3.9%, respectively. In this study the insufficient iodine intake was considered as median urinary iodine less than 150 μg/l in pregnant women [16].

All procedures performed in studies involving human participants were in accordance with the ethical standards of the institutional and/or national research committee and with the 1964 Helsinki declaration and its later amendments or comparable ethical standards.

### Statistical analysis

Normally distributed data are presented as mean ± standard deviation; non-normally distributed data are presented as median and interquartile range. Categorical data are presented as frequency and percentage. Departure from normality assumption was assessed by the Kolmogorov-Smirnov test. Chi-square or Fisher’s exact tests were used to show that whether the two categorical variables are independent. Between group differences were assessed using the t-test and ANOVA for normally distributed continuous data and Mann-Withney U test was also used in case on non-normal distributed data. To investigate the predictors of UIC deficiency and subsequent neonatal outcomes, multivariate binary and ordinal logistic regression models by the backward stepwise method by adjusting the probable confounders were undertaken. Analyses were performed using STATA 13 MP and a *p* < 0.05 was considered statistically significant.

## Results

The data from 884 mothers in their third trimester of pregnancy who had attended medical centres to terminate their pregnancies are used in this study. The mean ± standard deviation of the ages of the participants was 29.14 ± 2.5. Besides, the age pregnancy in the attending mothers were 38.4 ± 7.3 weeks gestational age. The median (IQR) urinary iodine concentration in the third trimester was 176 (165–196) micrograms/litre. Table 1 demonstrates the demographic information of the participants.

**Table 1:** Demographic information of studied participants based on iodine status

Variables	Urinary Iodine < 150 μg/l	Urinary Iodine ≥150 μg/l	All participants	*P*-value
	Mean(SD), N(%)	Mean(SD), N(%)	Mean(SD), N(%)	
**Urinary iodine concentration (**μg/l)	131.3 (130–137)	176 (165–197)	176 (165–196)	< 0.001
**Age (years)**	29.3 ± 4.9	29.2 ± 14.8	29.2 ± 14.5	0.91
**No. of live deliveries**	1.47 ± 0.89	1.73 ± 0.86	1.71 ± 0.87	0.091
**No. of previous pregnancies**	1.91 ± 0.91	2.07 ± 1	2.06 ± 1	0.25
**Weight before pregnancy (Kg)**	62.4 ± 11.2	64.5 ± 20.3	64.3 ± 19.9	0.25
**The timing between the two most recent pregnancies (year)**	3.25 ± 0.29	4.57 ± 0.15	4.5 ± 0.14	0.002
**Weight gain during pregnancy (Kg)**	9 ± 5	12.8 ± 5.5	12.6 ± 5.6	< 0.001
**Type of pregnancy**				
Planned	25 (4.0)	600 (96)	625 (72.93)	0.004
unplanned	21 (9.05)	211 (90.95)	232 (27.07)	
**Mother’s level of education**				
Illiterate	2 (8.3)	22 (91.7)	24 (2.72)	< 0.001
Senior high	27 (12)	198 (88)	225 (25.5)	
B.S.	16 (2.6)	606 (97.6)	622 (70.5)	
M.S. or higher	1 (9)	10 (91)	11 (1.25)	
**Iodinated salt consumption**				
yes	41 (5.2)	751 (94.8)	792 (89.59)	0.91
no	5 (5.4)	87 (94.6)	92 (10.41)	
**Complement intake during pregnancy**				
yes	32 (4)	775 (96)	807 (91.22)	< 0.001
No	14 (18.2)	63 (81.8)	77 (8.71)	
**Fish consumption**				
yes	34 (5.06)	638 (94.94)	672 (77.51)	0.54
No	12 (6.15)	183 (93.85)	195 (22.49)	
**Egg consumption**				
Twice weekly	30 (5.3)	539 (94.7)	569 (66.09)	0.93
Less than twice per week	15 (5.1)	277 (94.9)	292 (33.91)	

The results showed that out of 884 participants 838 (94.8%) had a urinary iodine concentration of more than 150 micrograms/litre and 46 (5.2%) showed urinary iodine concentrations less than 150 micrograms/litre. Assessment of the demographic variables of the two groups showed a statistically significance difference regarding the type of pregnancy (Planned vs. unPlanned), the use of dietary complements during pregnancy, the weight gained during pregnancy, the timing between the two most recent pregnancies and mother’s level of education. The results of multivariate logistic regression analysis used to assess the effects of possible variables influencing low urinary iodine concentration in the third trimester of pregnancy are shown in Table 2.

**Table 2:** Assessment of the effects of variables causing iodine urinary concentrations lowers than 150 micrograms/litre

Variables	Model 1^a^		Model 2^b^		Model 3^c^	
	(OR, 95% CI)	*P*-value	(AOR,95% CI)	*P*-value	(AOR,95% CI)	*P*-value
Age (years)	1.0 (0.98–1.02)	0.96	1.005 (0.98–1.02)	0.65	–	–
No. of live deliveries	0.67 (0.43–1.04)	0.079	0.85 (0.35–1.19)	0.24	–	–
No. of previous pregnancies	0.84 (0.61–1.15)	0.29	0.63 (0.30–1.35)	0.16	0.59 (0.39–0.89)	0.012
Weight gain during pregnancy	0.86 (0.8–0.92)	< 0.001	0.88 (0.82–0.95)	0.002	0.87 (0.83–0.95)	0.002
The timing between the recent two pregnancies	0.80 (0.68–0.94)	0.007	0.78 (0.64–0.95)	0.014	0.8 (0.68–0.95)	0.015
Type of pregnancy						
Planned	Reference	–	Reference	–	Reference	–
Unplanned	2.38 (1.30–4.35)	0.005	2.92 (1.29–6.58)	0.01	3.3 (1.65–6.63)	0.001
Mother’s level of education						
Illiterate	Reference	–	Reference	–	Reference	–
Senior high	0.88 (0.19–4.1)	0.88	0.22 (0.04–1.35)	0.11	–	–
M.S.	0.49 (0.1–2.1)	0.35	0.13 (0.3–0.73)	0.022	–	–
B.S or higher	1.1 (0.09–13.5)	0.94	0.1 (0.05–1.79)	0.11	–	–
Iodinated salt intake						
Yes	Reference	–	Reference	–	Reference	–
No	1.05 (0.40–2.73)	0.91	0.91 (0.11–2.29)	0.38	–	–
Complement intake during pregnancy						
Yes	Reference	–	Reference	–	Reference	–
No	5.38 (2.73–10.6)	< 0.001	3.64 (1.44–9.1)	0.006	4.8 (2.1–10.1)	< 0.001
Fish consumption						
Yes	Reference	–	Reference	–	Reference	–
No	0.81 (0.41–1.6)	0.54	0.89 (0.41–1.56)	0.58	–	–

^a^ univariate model

^b^ full model by enter method

^c^ backward stepwise model

The results of the aforementioned model show that every kilogram of weight gain during pregnancy has led to decreased odds of low urinary concentrations of iodine during the third pregnancy trimester of up to 13%. Moreover, with every one more year added to the time interval between the two most recent pregnancies led to a 20% of decrease of low urinary iodine concentrations. Besides, the odds of urinary iodine concentrations of lower the 150 micrograms/litre was 3.3 times more in those with unplanned pregnancies compared to those who experienced desired pregnancies. When assessed in accordance with the WHO criteria 46 of the participants (5.2%) had insufficient urinary iodine concentrations, 805 (91.06%) had adequate urinary iodine concentrations while 33 (3.73) showed more than adequate levels. There were no participants with urinary iodine concentrations higher than 500 micrograms/litre.

Assessing the neonatal complications showed that out of 884 neonates 29 (3.28%) suffered from severe neonatal hypothyroidism, 202 (22.85) had moderate hypothyroidism and the remainder had no dysfunction of the thyroid gland. The remainder of the maternal and neonatal complications are shown in Table 3.

**Table 3:** Association between neonatal complications and level of Iodine concentrations during the third trimester among pregnant women in Tehran

Type of complication	Category	UIC ≥ 150 μg/l (*n* = 838)	UIC < 150 μg/l (*n* = 46)	*P*-value^1, 2^
**Hypocalcaemia induced seizure**	**No**	822 (92.99)	46 (5.2)	0.34
	**Yes**	16 (1.81)	0 (0)	
**Neonatal hypothyroidism**	**No**	628 (71.04)	25 (2.83)	< 0.001
	**Moderate**	194 (21.95)	8 (0.9)	
	**Severe**	16 (1.81)	13 (1.47)	
**Anatomical congenital anomalies**	**No**	809 (91.5)	45 (5.09)	0.639
	**Yes**	29 (3.28)	1 (0.11)	
**Hyperbilirubinemia**	**No**	777 (87.9)	40 (4.52)	0.15
	**Yes**	61 (6.9)	6 (0.68)	
**RDS**	**No**	806 (91.18)	44 (4.98)	0.85
	**Yes**	32 (3.62)	2 (0.23)	
**NICU**	**No**	783 (88.57)	38 (4.3)	0.005
	**Yes**	55 (6.22)	8 (0.9)	
**Gestational age (week)**	**< 37**	96 (10.85)	12 (1.35)	0.008
	**≥37**	742 (83.9)	34 (3.84)	
**Apgar score at birth**	Mean (SD)	8.86 ± 0.6	8.82 ± 56	0.71^2^
**Apgar score at 5 min**	Mean (SD)	9.8 ± 1.2	9.7 ± 1.1	0.91^2^

1 Chi-square test 2 Independent t-test

This table demonstrates meaningful differences between the results of the univariate analyses concerning, preterm birth, NICU admission, neonatal hypothyroidism between the newborns of the mothers with urinary iodine concentrations higher than 150 micrograms/litre and those with lower concentrations. Besides, there was no significant relationship between the APGAR score at birth and urinary iodine concentration (0.11).

Table 4 shows the results of multivariate logistic regression performed to assess the relation of maternal urinary iodine concentrations and neonatal complications, adjusted for interfering variables. No relationship of significance was found between maternal urinary iodine concentrations and other outcomes.

**Table 4:** Multiple regression analysis of Iodine insufficiency effects on neonatal outcomes

Outcomes	AOR, 95% CI	*P*-value
NICU admission^1^	4.64 (1.81–11.9)	0.001
Preterm birth^2^	3.29 (1.51–7.1)	0.003
Neonatal hypothyroidism^3^	1.74^4^ (0.92–3.2)	0.083

1 This model was adjusted for maternal age, parity, pregnancy type, mother education, recent pregnancy time interval, weight gain during pregnancy, delivery type, smoking, Birth weight and Apgar score

2 This model was adjusted for maternal age, parity, pregnancy type, mother education, recent pregnancy time interval, weight gain during pregnancy and smoking

3 This model was adjusted for maternal age, parity, pregnancy type, mother education, recent pregnancy time interval, weight gain during pregnancy, delivery type, smoking and Birth weight

4 Derived from ordinal logistic regression

**Fig. 1:**
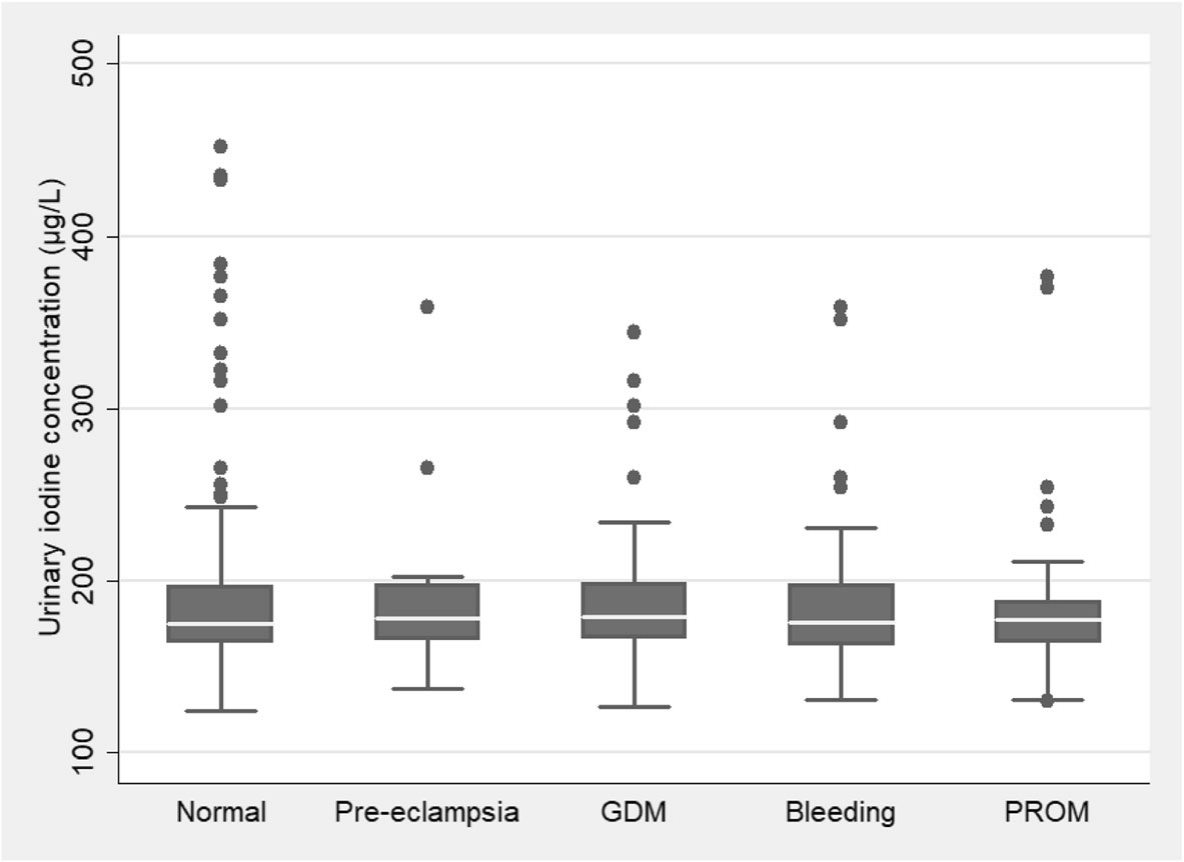
Mean level of urinary iodine concentration in different maternal complications

After adjusting the effects of confounding vaiables, the above-mentioned models showed that iodine deficiency in pregnant women has a strong relation with NICU admissions of the neonates and preterm births, so much so that inadequate urinary iodine concentrations led to a 4.6 times increase in NICU admissions and a 3.3 times increase in preterm births (results of full models along with all adjusted variables are available upon the request). The one way analysis of variance (ANOVA) showed that there are no statistical differences (*p* = 0.47) between the mean maternal urinary iodine concentration in different maternal complications of pregnancy (Fig. 1). Figure 1 showed the differences between the mean level of UIC in different maternal complications.

## Discussion

Our study demonstrated a prevalence of maternal iodine deficiency of 5.2%, as is defined by WHO, with a mean urinary iodine concentration of 131.3 (130–137) micrograms/litre. The main influencing factors on maternal iodine deficiency in this study were weight gain during pregnancy, the interval between the most recent pregnancies, whether or not the pregnancy has been Planned, mother’s level of education, and nutritional complement consumption. The need for a NICU admission, preterm birth and neonatal hypothyroidism were significantly related with maternal iodine deficiency before delivery.

According to WHO, ICCIDD and UNICEF guidelines there is no need for iodine complements in countries that iodinated salt is consistently consumed [17]. However, studies have shown that even in countries with consistent iodinated salt use 50% of pregnant women have urinary iodine concentrations below 150 micrograms/litre [18]. A recent study in Iran showed that the Iranian community has enough iodine intakes and has all of the IDD criteria for optimum control of iodine deficiency [19]. However, the same study showed that nearly 5% of the pregnant women participating in that study suffered from iodine deficiency. The results of a study in 2016 on the third-trimester iodine concentrations in pregnant women showed a 26.4% of urinary iodine concentrations below 150 and 28.7% below 100 micrograms/litre [20]. As this study took place in Tehran and in a specific group of pregnant women (third trimester), the differences in the prevalence can be attributed to the sampling methods and a much wider study environment..

Zimmermann et al. [21] carried out a study in European countries and found that iodine intakes during pregnancy were sufficient in ten countries and in 21 countries were insufficient. The results of a study conducted on 1525 mother-child pairs in the Netherlands revealed that 12.3% pregnant women had UIC < 150 μg/g creatinine in early pregnancy [22]. In another study on pregnant women in the rural area of Bangladesh, the prevalence of inadequate iodine intake was reported to be 6% and excessive iodine intake was 10% [23]. Zoysa et al. [24] in their study depicted that the prevalence of trimester-specific insufficient intake of urinary iodine in line with the World Health Organization criteria were 34.7, 45 and 62.2% in first, second, and third trimesters, respectively. These results were very similar to those of the present study, but the prevalence in the third trimester was less prevalent in the current study. Also, in Rajput et al.’ study [25], the median urinary iodine less than 150 μg/L among euthyroid pregnant women were 43.82, 54.08, and 62.24% in first, second, and third trimesters, respectively. Not only excessive iodine intake but also its deficiency can enhance the risk of goitre; based on a meta-analysis, a U-shaped association between UIC and goitre prevalence among school-age children can be observed [26].

Iodine deficiency during pregnancy has been one of the major problems in public health that affects developed and developing countries [27]. The importance of this issue arises from the fact that it affects the health of the mother and child and increases the financial burden related to this condition worldwide [28]. With effective interventions such as iodinating the dietary salt, educating pregnant women and proper coordination between medical and health departments, this problem can be prevented [29, 30]. Defining the causative chain of iodine deficiency during pregnancy and factors affecting it are necessary in order to prevent maternal iodine deficiency. In our study, there was a 13% decline with every one-kilogram weight gain during pregnancy. It appears that weight gain, as a proxy of proper diet, has protective activity against iodine deficiency [1]. Besides, there were meaningful relations between timing interval between most recent pregnancies, the desirability of the pregnancy, use nutritional micronutrients before and during pregnancy and mother’s level education and urinary iodine concentration. In a study in Ethiopia age, multiparity and mother’s level of education were found to be significantly related to maternal iodine content during pregnancy [31]. In another study in China the type of dietary salt, maternal age, occupation, consumption of complementary micronutrients and seafood were found to affect urinary iodine meaningfully [32]. In another study by Dineva et al. seafood consumption, iodinated salt intake and maternal age were found to be defining factors in urinary iodine concentration of the mother [33]. However, this study did not find any relation of statistical significance between maternal iodine deficiency and maternal complications of the pregnancy. Trolinska et al. did not find a relationship between iodine levels during pregnancy and maternal complications of the pregnancy, however, they proposed a larger study to assess the relationship [34].

The World Health Organization suggested the use of 250 mcg of daily iodine during pregnancy and lactation, and the United States Institute of Medicine (IOM) suggested 220 mcg of daily iodine during pregnancy and 290 mcg of daily iodine during lactation [17, 35]. There are associations between iodine deficiency during pregnancy and some complications including congenital anomalies, stillbirth, abortion, impair neurological development of the fetus, and irreversible fetal brain damage [36–39]. Geographic area, ethnicity, and different environmental factors affect the pattern of thyroid dysfunction. Thus, taking the importance of iodine in the health of the mother and the child into account, we must verify the urinary iodine status in various conditions.

Using a multivariate logistic regression in this study showed that neonate of mothers with urinary iodine levels below 150 micrograms/litre were 4.6 times more hospitalised in NICU and preterm outcome for the neonates of mothers with UIC below 150 micrograms/litre were 3.3 times more frequent in comparison with referent group. In a recent study in three major cities across the UK, researchers found no relation between iodine deficiency and low birth weight and preterm labour [40]. Walsh et al. did not find any relation between maternal low iodine intake and perinatal mortality and neural development of the neonates of these mothers [41].

Urinary iodine is considered as a non-invasive and efficient index for the population iodine status. Due to the fact that the majority of the absorbed iodine is excreted in the urine, urinary iodine can be regarded as a suitable indicator of recent iodine intake [42]. A single spot urine sample was used in this study. Single spot urine samples were recommended by WHO/UNICEF/ICCIDD for measuring the population iodine status [37]. Single spot urine samples in comparison with 24-h samples or multiple samples are easier in practice and based on a review, the results of spot urine samples are a valid indicator for measuring the population iodine status [43].

While this study with its large sample volume can be a good representation of the urban Tehran women, the existence of different racial minorities and ethnicities has created the need to repeat the study on other populations across the country. On the other hand, this study has focused on third-trimester mothers until delivery, while devising a study that includes the whole length of pregnancy can lead to a better assessment of the effects of maternal iodine concentrations during pregnancy on maternal and neonatal outcomes. Having had the thyroid function tests performed, we could have presented them as a complement to the current results.

## Conclusions

This study shows that despite implementation of iodized salt strategies in Iran approximately 5% of the pregnant population suffer from perinatal urinary iodine concentrations below 150 micrograms/litre. Iodine deficiency complication in pregnant women can be decreased by appropriate planning for pregnancy such as the planning for pregnancy, proper time intervals between pregnancies (> 12 months to < 5 years) [44, 45], appropriate nutrition during pregnancy. Besides, controlling maternal urinary iodine concentrations is important to prevent neonatal complications such as preterm delivery and NICU admission. Monitoring of iodine concentration at the population level and iodine replacement may be needed during pregnancy.

## Data Availability

All relevant data were available upon the reasonable request.

## Abbreviations

OR: Odd Ratios
AOR: Adjusted odds ratio
CI: Confidence Interval
NICU: Neonate Intensive care unit
RDS: Respiratory disease syndrome
PROM: Premature rapture of membrane
GDM: Gestational diabetes mellitus
